# The EIMB Hydrogel Microarray Technology: Thirty Years Later

**Published:** 2018

**Authors:** D. A. Gryadunov, B. L. Shaskolskiy, T. V. Nasedkina, A. Yu. Rubina, A. S. Zasedatelev

**Affiliations:** Engelhardt Institute of Molecular Biology, Russian Academy of Sciences, Vavilova Str., 32, Moscow, 119991, Russia

**Keywords:** hydrogel microarrays, nucleic acid hybridization, multiplex immunochemical assay, antimicrobial drug resistance, genotyping, tumor markers

## Abstract

Biological microarrays (biochips) are analytical tools that can be used to implement complex integrative genomic and proteomic approaches to the solution of problems of personalized medicine (e.g., patient examination in order to reveal the disease long before the manifestation of clinical symptoms, assess the severity of pathological or infectious processes, and choose a rational treatment). The efficiency of biochips is predicated on their ability to perform multiple parallel specific reactions and to allow one to study the interactions of biopolymer molecules, such as DNA, proteins, glycans, etc. One of the pioneers of microarray technology was the Engelhardt Institute of Molecular Biology of the Russian Academy of Sciences (EIMB), with its suggestion to immobilize molecular probes in the three-dimensional structure of a hydrophilic gel. Since the first experiments on sequencing by hybridization on oligonucleotide microarrays conducted some 30 years ago, the hydrogel microarrays designed at the EIMB have come a long and successful way from basic research to clinical laboratory diagnostics. This review discusses the key aspects of hydrogel microarray technology and a number of state-ofthe-art approaches for a multiplex analysis of DNA and the protein biomarkers of socially significant diseases, including the molecular genetic, immunological, and epidemiological aspects of pathogenesis.

## INTRODUCTION

Abundant knowledge on the molecular mechanisms of the biochemical processes that underlie the function of living systems has been accumulated over the past decade. This knowledge allows one to estimate the likelihood of someone developing a disease long before the manifestation of its clinical symptoms, to predict the severity of pathological or infectious processes, and to choose an effective and rational treatment. Solving the problems of personalized medicine should include both genome-wide analysis and the multiplex approaches used to quantify markers of pathological conditions.

Many approaches and techniques have been developed for the simultaneous, quantitative analysis of nucleic acid (NA) sequences. One such method, the microarray (biochip) technology, has proved efficient when used for transcription profiling, comparative genomic hybridization, and simultaneous identification of multiple targets in the genomes of humans, plants, microorganisms, and viruses [1]. The key component of a biochip platform is an array of spots, with each spot containing a probe whose nucleotide sequence is specific to a fragment of the analyzed genome. The reactions of NA hybridization and/or amplification performed simultaneously in each microarray element allow for parallel identification of different genomic targets, thus implementing the principle of multi-parameter analysis of a biological sample. Hence, DNA microarrays can be used as an efficient molecular tool to detect clinically significant markers of causative agents and the causes of socially consequential diseases

Microarrays can also contain matrixes of elements with immobilized proteins or oligosaccharides. Depending on the experimental objectives, each microarray element can carry either an individual, immobilized probe or their combination. The interactions between different classes of molecules involve a receptor–ligand, an antigen–antibody, an enzyme–substrate, and other types of interactions. When incubated with a specimen containing the molecules being analyzed, the immobilized ligand forms a specific complex. At this stage, a mixture of analyzed compounds is separated according to the ability of individual compounds to bind specifically to the immobilized ligands, making it possible to use a single microarray to simultaneously analyze different biological objects by implementing the principle of multiplex immunoassay. This test is required for proteomics research and for diagnosing diseases characterized by variations in many parameters in a patient’s serum.

## THE KEY ASPECTS OF A MICROARRAY ANALYSIS

A DNA microarray analysis is based on nucleic acid hybridization. The advantages of hybridization include its simplicity, multiplexity, and the reproducibility of results. Unlike enzymatic reactions, hybridization can be performed in a broad range of temperatures and buffer compositions. Meanwhile, nucleic acid hybridization does not allow for performing direct amplification of nucleic acids and must be used in combination with signal amplification methods or highly sensitive tools to detect nucleic acid duplexes. Therefore, microarrays are applied in direct quantification of RNA isolated from a large-volume specimen or for detecting the hybridization complexes formed by immobilized probes and the nucleic acid fragments obtained at the preliminary amplification stage. Hence, the sensitivity of a microarray assay depends on the initial amount of nucleic acids, amplification efficiency, and the method used to detect the complexes. The sensitivity of the most commonly used method - fluorescent detection of interactions in microarray elements - depends on the fluorescence analyzer.

In theory, DNA microarrays are supposed to ensure nucleic acid quantification [2]. However, real-world experiments show that there is significant quantitative bias in the gene expression data obtained using different microarray platforms and even different microarrays produced by the same manufacturer [3]. First, the hybridization kinetics nonlinearly depends on the density of the probes that reside on the microarray surface, since the oligonucleotides immobilized or synthesized on high-density microarray substrates are nonspecifically hybridized with each other, depending on their homology. Second, hybridization kinetics is affected by the length and nucleotide sequence of the target DNA molecules. Third, the quantum yield of a fluorophore used for detection depends both on the sequence of the adjacent nucleic acid and on proximity to other fluorophores. In this context, gene expression microarrays are used more often for reproducible analysis to evaluate the nucleic acid content rather than for an accurate determination of concentration [4].

One of the key parameters that characterize microarrays is the type of microarray substrate: substrates with hydrogel-based coatings (e.g., coatings made of polyacrylamide or agarose), as well as matrices carrying functional groups, such as aldehydes, epoxy or amino groups, etc. [5]. Due to their hydrophilic properties, polymeric hydrogels are high-priority substrate for biomolecule immobilization. The conventional approach to manufacturing microarrays consists in depositing a homogeneous hydrogel layer onto a substrate, followed by the immobilization of probes or *in situ *oligonucleotide synthesis. Both synthetic polymers (e.g., poly(2- hydroxyethyl methacrylate) and polyacrylamides) and non-synthetic polymer collagen are used as crosslinking agents to form hydrogel substrates [6]. As a result, the capacity of probe immobilization on microarrays increases by several orders of magnitude [7], making it possible to detect signals in microarray elements that are 10- to 100-fold more intense than the signals observed during immobilization on planar matrices.

A unique feature of microarray technology elaborated by researchers at the Engelhardt Institute of Molecular Biology, Russian Academy of Sciences, under the aegis of academician Andrey Darievich Mirzabekov (1937–2003), is the immobilization of molecular probes in 3D hydrogel elements anchored to a planar substrate [8, 9].

**Fig. 1:**
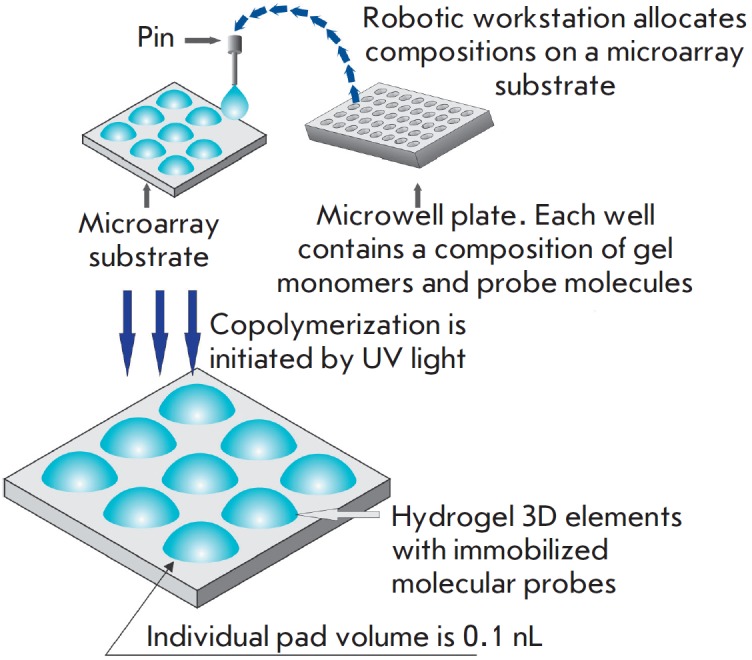
Manufacture of a microarray with 3D elements containing compositions of hydrogel and molecular probes

Molecular probes, oligonucleotides or oligosaccharides are modified by attachment of amino- or sulfo groups that are subsequently used as covalent binding sites during chain propagation. Meanwhile, protein-based probes require no special modification, as they carry proper functional amino acid groups within their structure. The macroporous structure of hydrogel elements is formed via copolymerization of a methacrylic acid derivative monomer with an unsaturated derivative of O- or N-substituted saccharide, a bifunctional crosslinking agent, and a molecular probe. The polymerization mixture (0.1 nL) is applied onto a substrate by the pins of a robotic workstation (*Fig. 1*). Almost any carriers (glass, plastic, etc.) can be used as a substrate. Next, UV radiation-induced copolymerization of molecular probes with the main hydrogel components takes place and the compounds being immobilized are uniformly embedded into the growing polymer structure. It should be mentioned that optimal conditions for molecular probe polymerization have been selected, making it possible to maximally retain the original biological activity of the probes. After polymerization, hydrogel elements (pads) formed on the substrate are washed and prepared for experiments. The efficiency of this immobilization procedure is 50–80%, depending on the particular molecular probe.

Depending on the type of microarray, the diameter of the gel element varies from 50 to 300 μm; the distance between pads can range between 100 and 500 μm. The number of spots per microarray depends on the specific task that needs to be solved and varies from several dozens to several thousands. The application quality is controlled by a specialized hardware and software complex. Microarrays in which the discrepancy in the geometric parameters of the elements is ≤ 10% and the discrepancy in the parameters between different microarray batches is ≤ 20% are used for further experiments [10]. These characteristics comply with the criteria used for the best commercial microarrays, manufactured by ArrayIt (USA) and Schott AG (Germany).

**Fig. 2:**
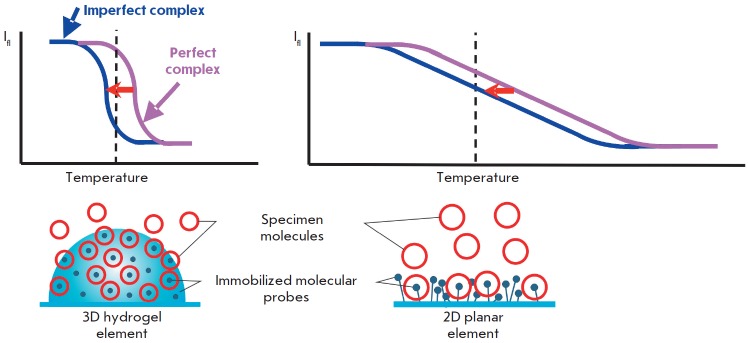
Advantages of biochips with 3D elements in comparison with 2D elements residing on a planar surface. Molecular complexes formed in 3D elements and distributed uniformly throughout the volume are located in the water-like homogeneous environment of the hydrogel and have identical energies of formation. In this case, the temperature-induced dissociation of such complexes occurs in a narrow temperature range and it is always possible to select a temperature at which the perfect complex remains stable, while the imperfect complex will be substantially dissociated. Therefore, in the case of 3D elements, perfect complexes can be detected by signals exceeding the signals of imperfect complexes several times. In 2D elements, the energy of complex formation is superimposed with different energy interactions between the complexes and the substrate surface. As a result, the dissociation curves of molecular complexes have a gentle slope and the temperature shift (usually 3–4°C) between the curves characterizing the perfect and imperfect complexes is insufficient to provide a significant difference in the corresponding signals

Since the first experiments on sequencing by hybridization with oligonucleotide micromatrices conducted some 30 years ago [11], hydrogel microarrays have come a long way from basic research to clinical laboratory diagnostics. Kinetic and thermodynamic studies of hybridization of DNA fragments have demonstrated that application of short probes (up to 25 nucleotides long) make it possible to efficiently discriminate between point nucleotide substitutions, while immobilization in 3D hydrogel elements significantly enhances the intensity of positive signals and reduces the statistical dispersion, as compared to 2D planar microarrays [12, 13] (*Fig. 2*).

Gryadunov *et al*. [14] experimentally selected the hybridization conditions and concentrations of immobilized probes; they also proposed algorithms for computing probe sequences that would ensure high positive signals and discrimination ratios. Substantial progress was made in the analytical sensitivity of the assay thanks to the elaboration of a multiplex PCR assay procedure that can simultaneously amplify several dozen genome fragments [15, 16], as well as thanks to the synthesis of novel dyes and the optimization of fluorescent labeling [17, 18].

Rubina *et al. *[19] have developed, for the first time, procedures for efficient immobilization of protein and glycan molecules in hydrogel and proposed methods for multiplex quantitation of different proteins in serum. Several generations of universal fluorescence analyzers have been designed. The most recent one can be used to measure the signal intensity of microarray elements at wavelengths ranging from 380 to 850 nm and perform qualitative and quantitative analyses with an accuracy of ± 5% [20].

The universal platform of hydrogel microarrays designed by EIMB researchers has made it possible to elaborate, validate, and implement a number of applications for multi-parameter analysis of the biomarkers of socially important diseases. These applications will be discussed below.

## ANALYSIS OF SPECIFIC SEQUENCES OF BACTERIAL AND VIRAL GENOMES

The need to analyze bacterial and viral genomes is largely rooted in the social importance of pathogens, which often include the drug-resistant causative agents of tuberculosis (*Mycobacterium tuberculosis*, MTB) and mycobacteriosis (non-tuberculous mycobacteria, NTM), hepatitis C virus (HCV), and microorganisms belonging to the group responsible for infection and inflammation of the reproductive tract. With regard to these objects, the technology of hydrogel-based DNA microarrays has proved to be an efficient tool for determining the profile of antibiotic resistance determinants, as well as for intra- and interspecies genotyping of microorganisms and viruses in order to select an adequate therapy and perform epidemiological surveillance.

**Application of microarrays in the laboratory diagnosis of tuberculosis**

The TB-Biochip-1 diagnostic kit for identifying 48 mutations in the *Mycobacterium tuberculosis *genome, which are responsible for the resistance of this bacteria to rifampin (RMP) and isoniazide (INH), was the first microarray-based assay in the world to be designed and approved for *in vitro *clinical diagnostics [21]. The diagnostic characteristics of this method were evaluated using the results of a 10-year (2005–2015) application of the TB-Biochip-1 kit in medical anti-tuberculosis institutions in Russia, Kyrgyzstan, and Azerbaijan. A meta-analysis of 16 publications that reported data on an evaluation of > 5,000 clinical specimens and isolates using a TB-Biochip-1 diagnostic kit and microbiological assays demonstrated that the diagnostic sensitivity of this method, used to identify the RMP-resistant phenotype of MTB, lies in the 88.8–96.9% range and that its specificity is 90.3–99.4%. When this method is used to analyze INH-resistant strains, its sensitivity and specificity are 85.7–96.9% and 85.3–98.2%, respectively. There was an 80–90% match between the results obtained using the TB-Biochip-1 kit and molecular assays recommended by the WHO (Xpert MTB/RIF (Cepheid, USA) and Genotype MTBRD*plus *(Hain Lifescence, Germany)) [22, 23].

An important result is that the TB-Biochip-1 kit proved effective in the treatment of patients with destructive pulmonary tuberculosis, depending on the time when the treatment schedule was adjusted, as confirmed by the chief visiting Physiologist of the Ministry of Health of the Russian Federation [24]. When using microarrays for early diagnosis of multidrug-resistant (MDR) forms of tuberculosis, the number of cured patients increased at least threefold, as opposed to the case when the diagnosis was made using standard culture-based methods [14, 25]. Today, the TB-Biochip-1 kit is still actively used for laboratory diagnostics of tuberculosis. It promptly reveals multidrug-resistant isolates, so the patients can be switched to other treatment schedules.

**Fig. 3:**
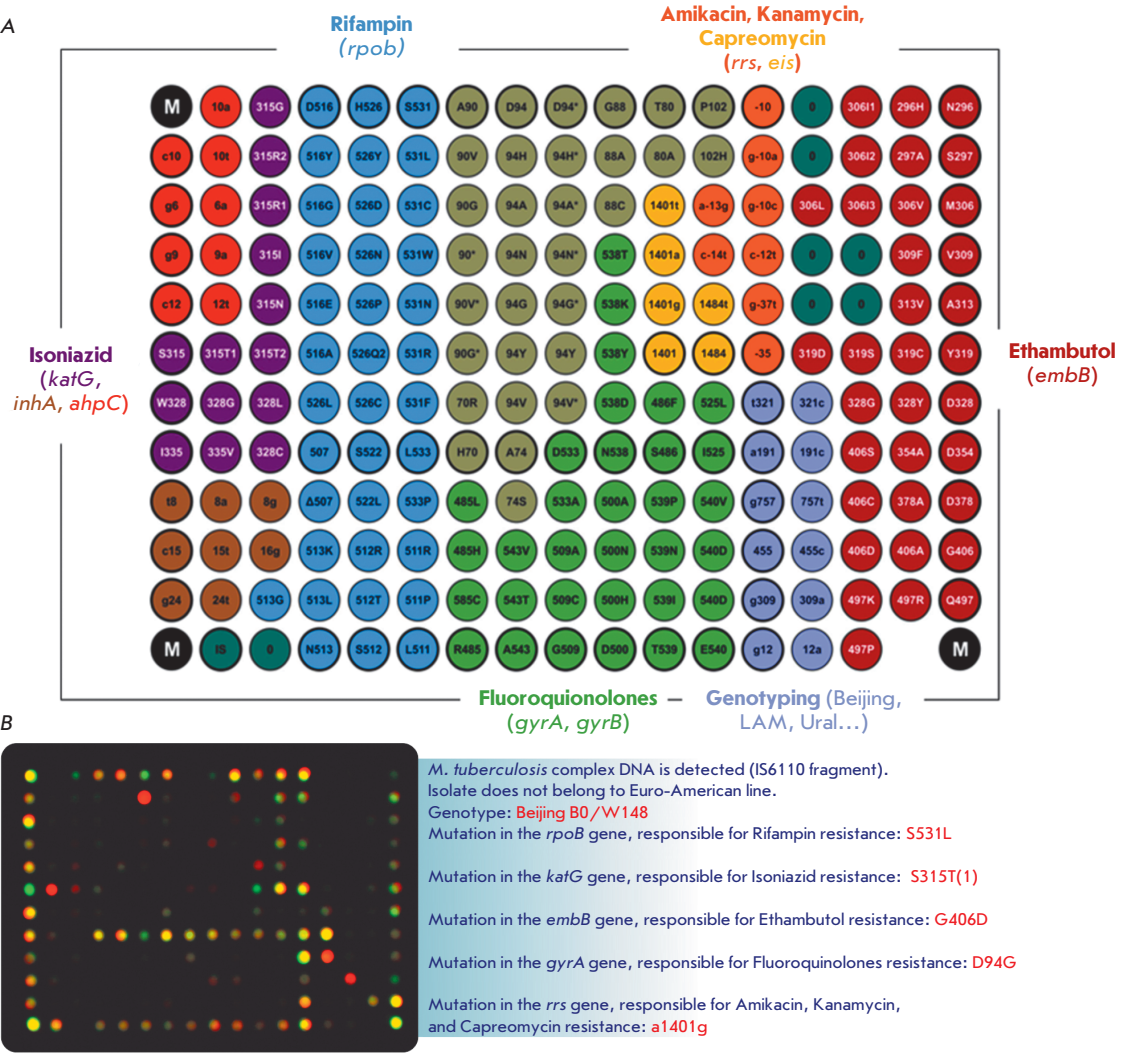
(*A*) Microarray configuration for simultaneous determination of the MTB genotype and identification of the MDR and XDR genetic determinants. Various colors show groups of elements with immobilized probes specific to wild-type sequences and mutant variants of the genes associated with drug resistance to different anti-tuberculosis drugs. (*B*) An example of the biochip hybridization pattern and the result of interpretation upon analysis of *M. tuberculosis *DNA from an extensively drug-resistant isolate of the Beijing B0/W148 genotype

Meanwhile, sequential accumulation of mutations associated with drug resistance not only increased the number of incident patients with MDR forms of tuberculosis (from ~15% in 2005 [21] to ~50% in 2015 [26]), but also resulted in the emergence of isolates with extensive drug resistance (XDR) and total drug resistance to all antituberculosis medication. In order to solve these problems, we have developed a method that allows one to detect MTB DNA and simultaneously identify the genotype of strains endemic to Russia and genetic determinants of MDR and XDR. The analysis procedure includes multiplex PCR assay with adapter primers and cyclic elongation to ensure simultaneous amplification and labeling of 17 loci of the *M. tuberculosis *genome, followed by hybridization. As the key component of this approach, the microarray allows one to identify MTB DNA, to determine the lineages of the causative agent endemic to Russia, and to detect a total of 116 genetic determinants of drug resistance to rifampin, isoniazid, fluoroquinolones, injectable drugs (amikacin, kanamycin, and capreomycin), and ethambutol (EMB) (*Fig. 3*).

A clinical trial of the method conducted at the St. Petersburg Research Institute of Phthisiopulmonology of the Ministry of Health of the Russian Federation dem onstrated that the diagnostic sensitivity and specificity of the elaborated procedure amounted to > 90% for all drugs, except for ethambutol [15]. Sensitivity for this drug was 89.9%, significantly higher than the value published earlier (58%) [27].

**Fig. 4:**
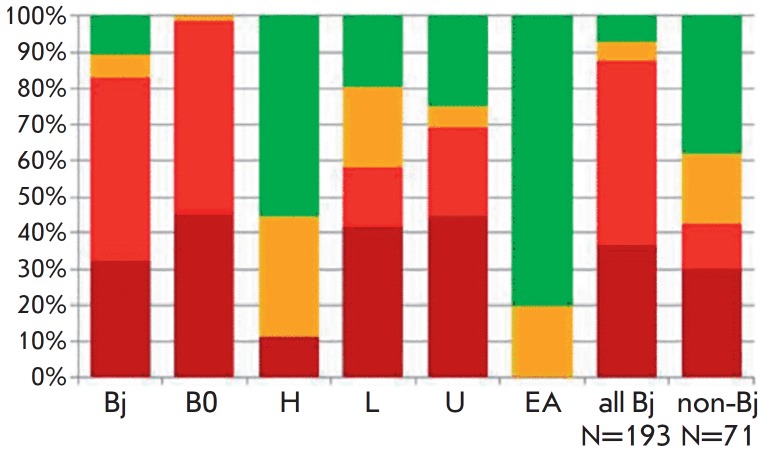
Association of MTB lineages with drug resistance. The drug resistance profile is marked with colors: dark red – XDR, red – MDR, yellow – mono- or poly-drug-resistant, green – susceptible isolates. Designations of lineages: Bj – Beijing; B0 – Beijing B0/W148; H – Haarlem; L – LAM; U – Ural; EA – Euro-American lineage

An analysis of MTB lineages revealed that strains with the Beijing genotype were predominant (73.1%). The LAM (12.1%) and Ural (~7%) families, as well as Euro-American strains (7.2%), were less frequent (*Fig. 4*). The B0/W148 cluster accounted for > 30% of all Beijing genotype isolates. If an isolate was found to belong to a certain genotype, this meant that the MDR or XDR phenotype was revealed with a 100% probability, thus confirming the clinical significance of the detection of this genotype. Contrariwise, isolates of the Euro-American lineage not belonging to the LAM and Ural families were mostly associated with the drug-susceptible phenotype [15].

Nosova *et al*., in collaboration with the Moscow Research and Clinical Center for Tuberculosis Control of the Moscow Government Health Department, revealed correlations between the genetic determinants of drug resistance and minimal inhibitory concentrations that characterize the level of resistance to a certain anti-tuberculosis drug [28]. It is very important that the results of the analysis of the determinants associated with different resistance levels allow physicians to prescribe different doses of anti-tuberculosis drugs belonging to an extremely narrow range of specific therapeutic agents.

The elaborated method has underlain the development of a TB-TEST diagnostic kit. The TB-TEST kit has undergone trials and has been approved for use by the Russian Federal Service for Surveillance in Healthcare and Social Development. The TB-Biochip diagnostic kits are giving way to the application of the TB-TEST kit. The range of genomic targets for first-and second-line drugs that can be analyzed using the TB-TEST kit includes at least chemotherapy schedules I–IV for tuberculosis patients in compliance with Order no. 951 of the Ministry of Health of the Russian Federation dated December 29, 2014. The speed and feasibility of the analysis of respiratory material allow clinicians to use this kit for rapid screening of patients’ specimens and subsequent adjustment of therapy schedules and switching of patients to new anti-tuberculosis drugs [29].

The SPOLIGO-BIOCHIP kit has been developed for routine intraspecies genotyping of strains of the Mycobacterium tuberculosis complex. This kit provides information about the genetic profile of each MTB isolate by identifying its genotype [30]. The approach is also used to differentiate between MTB and the tuberculosis vaccine strain *M. bovis BCG *in contents of cold abscess in children with post-vaccination complications.

The species-specific polymorphism of the *gyrB *gene in microorganisms belonging to the *Mycobacterium *genus made it possible to design a microarray for identifying 35 different mycobacterial species [31]. An analysis of mycobacterial populations in the Central and Northwestern regions of the Russian Federation revealed that such NTM species as the *Mycobacterium avium *complex (39%), *M. fortuitum *(17%), and *M. xenopi *(13%) predominate in European Russia. The infection caused by these NTM species manifests itself in immunosuppressed patients, as well as patients with a chronic obstructive pulmonary disease and HIV [31].

Hence, the combination of diagnostic kits in the analysis of the causative agents of tuberculosis and mycobacteriosis allows one to thoroughly examine material collected from patients using a universal diagnostic microarray platform in a clinical laboratory. A unified assay that complies with all current requirements, automated analysis of the results, and their interpretation through the provision of specific recommendations allow one to improve the treatment schedules of tuberculosis caused by drug-resistant strains.

**Analysis of the genetic determinants of antibiotic resistance of the causative agents of reproductive tract infections**

There are significant challenges that are related to the diagnostics and selection of a personalized therapy strategy for reproductive tract infections (RTIs) due to the wide variety of RTIs that often develop as mixed drug-resistant forms, including both sexually transmitted obligate pathogens and a number of causative agents of opportunistic infections. *Neisseria gonorrhoeae *holds a special place among the causative agents of RTIs. Similar to the causative agent of tuberculosis, gonococci possess an extraordinary ability to develop drug resistance. Unlike in MTB, chromosomal mutations are not the only mechanism through which *N. gonorrhoeae *acquires new resistant properties . It also actively employs various mobile genetic elements and horizontal gene transfer from other species. The mutations affecting membrane permeability and enhancing efflux pump activity are especially efficient in *Neisseria gonorrhoeae*, since these systems can help simultaneously develop resistance to many antimicrobials [32].

**Fig. 5:**
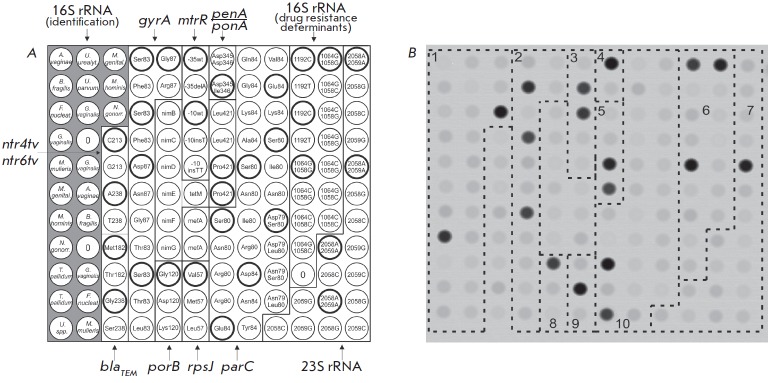
(*A*) Microarray for the analysis of the drug resistance of RTI causative agents. The biochip contained immobilized probes corresponding to the species-specific polymorphism of the *16S rRNA *gene, which were used for the identification of microorganisms, and also probes specific to the *rrs*, *rrl*, *gyrA*, *parC*, *mefA*, *mtrR*, *nimB-G*, *penA*, *ponA*, *porB*, *rpsJ*, *ntr4tv*, *ntr6tv*, *blaSHV*, *blaTEM*, and *tetM *genes sequences, which act as determinants of resistance of RTI causative agents to different AMD. The elements containing wild-type oligonucleotides are circled in black. (*B*) The hybridization pattern obtained by analyzing *N. gonorrhoeae *DNA contained the following mutations: S91F+D95G in the *gyrA *gene (group 2), -35delA in the promoter of the *mtrR *gene (group 3), insD345 in the *penA *gene (group 4), and S87R in the *parC *gene (group 10)

A microarray (*Fig. 5A*) and a procedure for its use have been developed to identify the DNAs of 12 different obligate and opportunistic microorganisms and simultaneously perform the differential analysis of 39 genetic determinants of resistance to β-lactam antibiotics, macrolides, aminoglycosides, tetracycline, spectinomycin, fluoroquinolones, and nitroimidazole [33].

An analysis of more than 500 clinical specimens and isolates obtained at the State Research Center of Dermatovenerology and Cosmetology of the Ministry of Health of the Russian Federation has demonstrated that the developed method exhibits high sensitivity and specificity in the identification of the DNA of the causative agents of RTIs. It also allowed clinicians to determine prognostic efficiency in identifying the markers of antibiotic resistance for these agents.

A study focused on tetracycline-resistant strains of *N. gonorrhoeae *isolated in Russia in 2015–2017 demonstrated that long-term interruption (since 2003) in the use of tetracycline for the treatment of gonorrhea led to a reduction in the percentage of resistant strains in Russia ranging from 70 to 42.6%. However, this does not provide grounds for recommending tetracyclines for the treatment of gonococcal infection. The type of *tetM *gene in plasmid DNA associated with a high level of tetracycline resistance regardless of the presence of chromosomal resistance determinants was characterized for the first time in Russia [34].

**Fig. 6:**
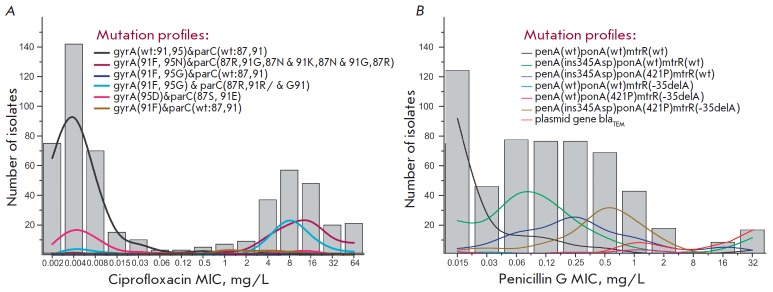
The distribution of *N. gonorrhoeae *isolates with different mutation profiles for minimal inhibitory concentrations (MICs) of ciprofloxacin (*A*) and penicillin G (*B*). (*A*) Profiles include mutations in the *gyrA *and *parC *genes, leading to resistance to ciprofloxacin. (*B*) Profiles include mutations in the *penA, ponA, *and *mtrR *genes associated with resistance to penicillin G. The profile of isolates carrying the *bla_TEM_*plasmid gene is shown separately. Designation: wt – wild type

The *N. gonorrhoeae *strains carried multiple mutations in the *penA*, *ponA*, *rpsJ*, *gyrA*, *parC*, *mtrR*, and other genes (*Fig. 5B*). The prognostic significance of these mutations with respect to phenotypic resistance substantially increased in the presence of combinations of genetic resistance determinants (*Fig. 6*) [35, 36].

This circumstance is in definite conflict with the fact that modern therapy for gonococcal infection is based on a preferential use of third-generation cephalosporins and technically does not exert “selection pressure” on the genetic determinants that regulate resistance to the drugs used earlier (penicillins or fluoroquinolones). Hence, it is reasonable to expect these mutations to be eliminated from the genome of the modern population of *N. gonorrhoeae*. The presence of these mutations could be attributed to the multifactorial nature of antibiotic resistance, where a number of earlier gene mutations underlie the next turn of the evolutionary spiral of *N. gonorrhoeae*. In particular, this is true for the *penA *and *ponA *genes, whose mutations currently appear to be significant for the developing resistance to cephalosporins. Hence, it is fair to anticipate an emergence of resistance to modern antibiotics (first of all, among the multidrug-resistant strains of *N. gonorrhoeae *as is happening in EU member states) [37]. This circumstance is an indication that continuous monitoring of antibiotic resistance by the causative agent of gonorrhea is a rather topical issue. The hydrogel microarray technology is currently one of the methods used for such monitoring.

**Identification of the genotype and subtype of the hepatitis C virus by analyzing the NS5B region of the viral genome**

**Fig. 7:**
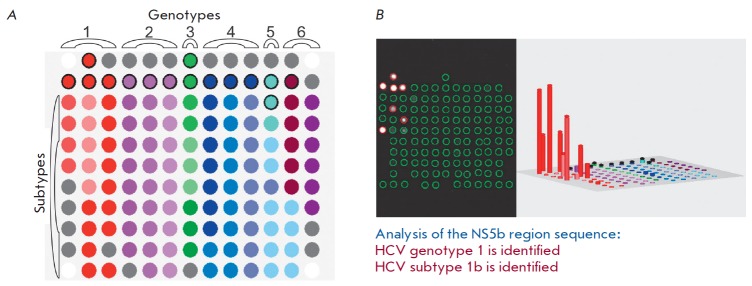
(*A*) Configuration of microarray with 110 immobilized oligonucleotides for identifying the genotypes and subtypes of HCV. Elements containing genotype-specific probes in the upper rows are encircled in a thick black contour. (*B*) The hybridization pattern of HCV RNA subtype 1b, histogram of signal intensities of biochip elements, and results of interpretation

According to existing classification, the hepatitis C virus (HCV) is subdivided into seven main genotypes and 67 subtypes [38]. The HCV genotype and subtype are the key determinants taken into account when choosing schedules of treatment with direct-acting antiviral agents (DAAs) affecting the key targets of the virus life cycle [39]. Accurate identification of the HCV genotype and subtype defines the choice of treatment schedule (particular DAA, treatment course duration, and whether or not ribavirin needs to be prescribed).

In collaboration with the Virology Laboratory of the University of Toulouse (France), researchers have proposed a method for identifying six genotypes and 36 subtypes of HCV by analyzing the genotype- and subtype-specific sequences in the HCV NS5B on a hydrogel microarray (*Fig. 7A*). The analysis procedure involves amplification and fluorescent labeling of the NS5B region, followed by hybridization on microarray, signal detection, and interpretation. An example of the assay for a HCV subtype 1b RNA sample and interpretation of the results are shown in *Fig. 7B*.

The developed method was used to analyze 345 HCV specimens as compared to the “gold standard” of genotyping, namely, sequencing of the NS5B region, followed by a phylogenetic analysis. The genotypes were identified for all RNA samples with 100% match. Matching results of subtype identification were obtained for 329 out of 330 specimens [40].

The characteristics of the designed HCV-Biochip kit render it as efficient as the sequencing technology. Being an efficient tool for routine genotyping, this method can be used to evaluate the response to treatment with DAAs, depending on the HCV subtype [41, 42].

## MICROARRAYS FOR PERSONALIZED TREATMENT OF CANCER PATIENTS

**Molecular genetic analysis of chimeric genes in leukemia**

Detection of structural genomic rearrangements that give rise to chimeric genes in tumor cells in the bone marrow (especially in children) is used in most state-of-the-art protocols to divide patients into risk groups and to choose the proper therapy.

**Fig. 8:**
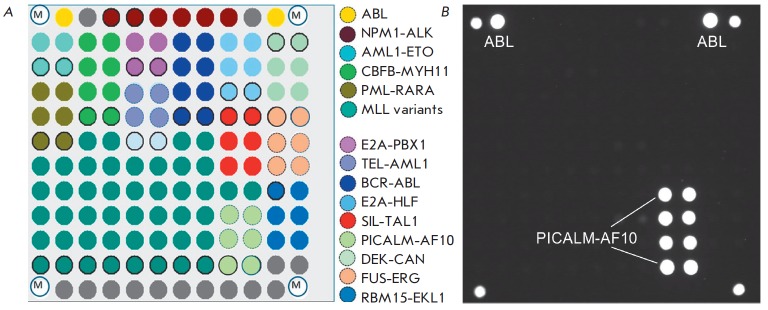
(*A*) Microarray layout for the identification of the chromosomal rearrangements leading to different types of leukemia. Elements of the biochip with immobilized oligonucleotides specific to the sequences of different chimeric genes are marked in various colors. (*B*) The hybridization pattern obtained by analyzing an RNA sample containing the PICALM-AF10 fusion transcript that is associated with a poor prognosis. Such type of leukemia requires allogeneic transplantation of hematopoietic stem cells

A LK-BIOCHIP kit has been developed and approved by the Russian Federal Service for Surveillance in Healthcare and Social Development for the analysis of the 13 most clinically significant chromosomal breakpoints in leukemia [43]. The LK-BIOCHIP was used to diagnose chromosomal translocations in children in multi-center trials aimed at treating acute lymphoblastic leukemia (ALL MB-2002 and ALL MB-2008) in the Russian Federation in 2005–2015 [44]. The new generation of microarrays for leukemia diagnosis is capable of detecting an extended range of chromosomal translocations, including nine additional clinically significant rearrangements t(1;11) MLL/MLLT11, t(1;11) MLL/EPS15, Del1 SIL/TAL1, t(2;5) NPM1/ALK, t(16;21) FUS/ERG, t(1;22) RBM15-MKL1, t(10;11) CALM/AF10, t(17;19) E2A/HLF, and t(6;9) DEK/CAN (*Fig. 8*). The diagnostic kit can detect one tumor cell among 1,000 normal ones in a clinical specimen, with a specificity ≥ 95% [45].

**Microarrays for analyzing somatic mutations**

Detection of somatic mutations in tumor tissue allows one to choose specific drugs that engage the desired molecular targets for treatment. The proportion of mutant DNA in the analyzed material is often negligible because of tumor heterogeneity or contamination of the specimen with normal tissue. Paraffin-embedded tumor tissue blocks are typically used as material for molecular genetic examination. When stored under these conditions, tumor DNA is partially degraded and fragmented; so, there are some limitations associated with the application of molecular genetic methods.

**Fig. 9:**
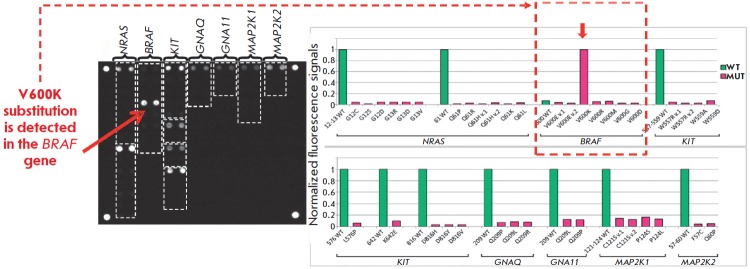
Hybridization pattern and histograms of normalized signals of biochip elements for the determination of somatic mutations in DNA samples of skin melanoma. The V600K substitution in the *BRAF *gene is detected. Administration of the BRAF inhibitors vemurafenib and dabrafenib is recommended

Emelyanova *et al*. [46] developed a method for analyzing somatic mutations using a microarray; the detection limit for revealing mutant DNA reached 0.5%. This approach was used to analyze somatic mutations in patients with melanoma. The recent breakthrough in the treatment of this disease is associated with the application of targeted drugs that specifically act on the molecular targets and immunotherapy, whose effectiveness largely depends on the tumor genotype. This method relies upon using a microarray to identify the 39 clinically relevant somatic mutations in the *BRAF, NRAS, KIT, GNAQ, GNA11, MAP2K1*, and *MAP2K2 *genes (*Fig. 9*).

A total of 253 clinical specimens of melanoma were tested using this method. Various mutations in the *BRAF *(51.0%), *NRAS *(17.8%), *KIT *(2.4%), *GNAQ *(1.6%), *GNA11 *(0.8%), and *MAP2K1 *genes have been revealed (0.8%). The approach allows one to efficiently detect clinically relevant somatic mutations and choose the optimal target drug in 70% of melanoma patients [47].

## MULTIPLEX IMMUNOASSAY USING MICROARRAYS

Depending on the specific clinical problem, there are two main types of multiplex immunoassays: (1) identification of various individual antigens in the specimen or (2) detection of antibodies circulating in the serum. In the former case, the microarray contains a panel of immobilized antibodies and each of these antibodies specifically binds to a certain antigen under analysis. In the latter case, the microarray contains immobilized ligands of protein or other nature, which specifically bind to immunoglobulins within the specimen. An example of the type 1 method is the kit for the quantitation of a number of biotoxins developed in collaboration with researchers from the M.M. Shemyakin– Yu.A. Ovchinnikov Institute of Bioorganic Chemistry of the Russian Academy of Sciences (IBCh RAS), under the aegis of academician E.V. Grishin [48].

**Multiplex microarray analysis of tumor markers**

A large number of studies have focused on a search for clinically significant biomarkers exhibiting high sensitivity and specificity with respect to certain tumors. The diagnostic efficiency can be enhanced by simultaneous detection of several tumor markers.

Meanwhile, some tumors cannot be timely detected using this approach. Hence, an analysis of the serum tumor markers CEA and CA 19-9 is recommended for *in vitro *diagnosis of colorectal cancer (CRC). The results of large-scale clinical trials demonstrate that these biomarkers mostly detect the disease at its late stages (III and IV) and are clinically relevant only for on-treatment monitoring [49]. Most CRC-associated markers are glycoproteins or carbohydrate antigens; they contain either O- or N-glycosites [50]. Modification of the glycosylation of these markers changes the levels of respective antigens, which can be detected by multiplex microarray assay.

**Fig. 10:**
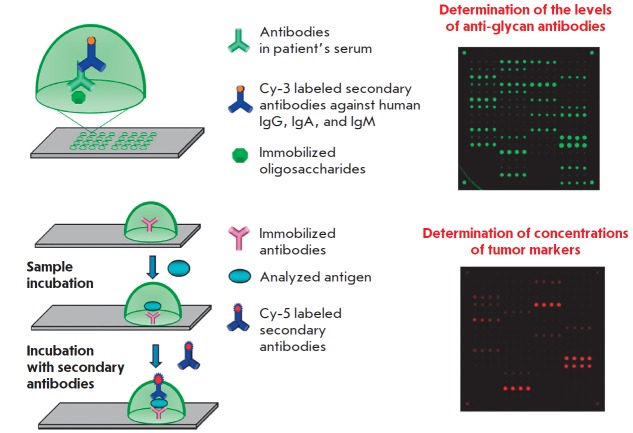
Simultaneous determination of the levels of antibodies against tumor-associated glycans and concentrations of tumor markers. An immunoassay scheme and an example of fluorescence biochip images after analysis are shown

An approach relying on a simultaneous analysis of serological protein-based tumor markers and antibodies belonging to different classes specific to tumor-associated glycans has been developed in collaboration with researchers at the Laboratory of Carbohydrates of the IBCh RAS. A combined microarray has been designed, with its elements containing glycans and antibodies specific to tumor markers for CRC. The serum levels of anti-glycan antibodies were determined by fluorescent microarray assay (*Fig. 10*).

Studies carried out in collaboration with the P.A. Hertzen Moscow Cancer Research Institute revealed combinations (signatures) consisting of levels of antibodies against some tumor-associated glycans and concentrations of the main tumor markers, which made it possible to reliably differentiate between CRC patients and healthy volunteers [51]. It has been demonstrated that combined use of protein and glycan signatures has a better predictive value for detecting CRC than the conventional pair of CEA + CA 19-9 tumor markers. The sensitivity and specificity of this method were 88% and 98, respectively, while the combination of CEA and CA 19-9 detected CRC in 80% of the cases with 21% sensitivity.

**Microarray analysis of allergen-specific immunoglobulins (Igs)**

Today, the key markers of allergy are immunoglobulins E, which mediate type I allergic reactions (anaphylaxis and Quincke’s edema). Immunoglobulins belonging to the other classes can also be involved in allergic reactions. Thus, specific immunoglobulins belonging to the IgG4 class (sIgG4) play a role in the development of tolerance (i.e., the absence of clinical manifestations in response to certain allergens, following sensitization to these allergens). Although sIgG4 is not a diagnostic marker, identification of this parameter is important for evaluating the sIgE/sIgG4 ratio, which shows the effectiveness of a specific immunotherapy. sIgG4 act as blocking antibodies impeding the development of type I allergic reactions [52].

**Fig. 11:**
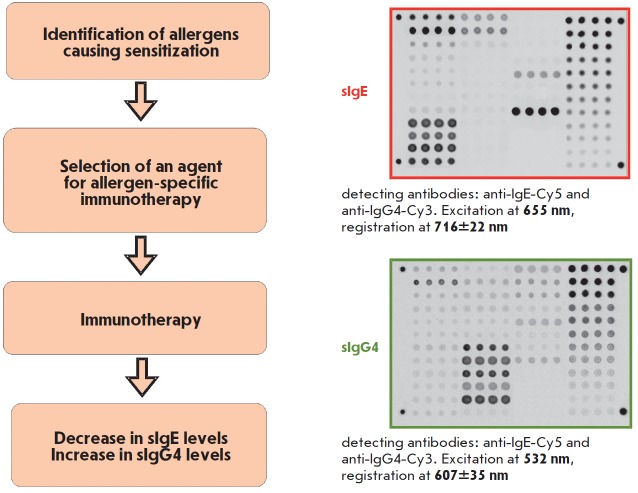
Multiplex analysis of the sIgE and sIgG4 panels for the diagnosis and monitoring of allergy therapy. Fluorescent images of the same biochip were presented after a serum sample was analyzed

The Allergobiochip kit (*Fig. 11*) designed at the EIMB in collaboration with DR. FOOKE Laboratorien GmbH company (Germany) is intended for a parallel analysis of sIgE and sIgG4 panels specific against the following classes of allergens: pollen allergens (trees and shrubs; weeds and flowers; grasses and graminaceous plants), epidermal allergens, insect venom allergens, mite allergens, food, and fungal allergens. The diagnostic kit is a modification of solid-phase immunoassay involving fluorescent signal detection on the microarray platform. This method was tested using more than 2,000 serum specimens collected from allergic patients and healthy volunteers. The Allergobiochip kit has proved efficient in detecting type 1 hypersensitivity. Detection thresholds for sIgE and sIgG4, as well as sensitivity and specificity of the assay, were determined. The measurement range was 0.35–100 IU/mL for sIgE and 100–2500 ng/mL for sIgG4 [53, 54].

**Fig. 12:**
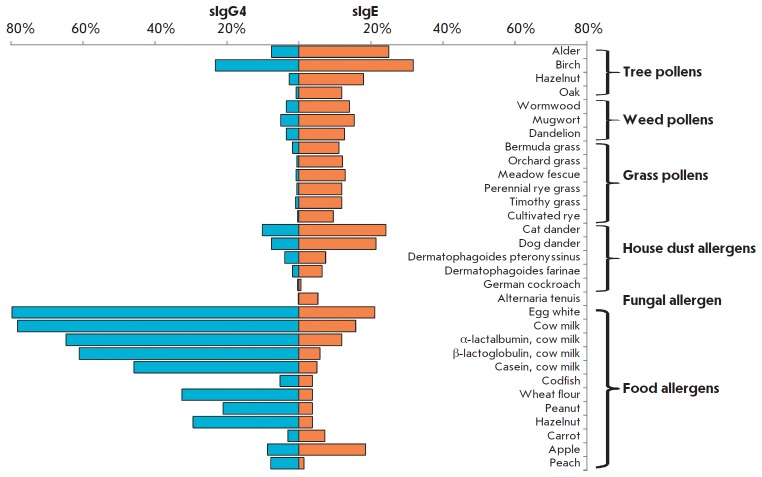
Analysis of the frequency of allergic sensitization of different groups in allergic diseases. The percentages of patients (%) aged 0–16 years from the Moscow region with allergic symptoms and increased concentrations of sIgE (≥ 0.35 IU/mL) and sIgG4 (≥100 ng/mL) to each of the 31 allergens

An epidemiological study involving a model pediatric population residing in central Russia (800 patients at the Filatov Moscow Pediatric Clinical Hospital aged 0–16 years and 50 healthy volunteers) was conducted to evaluate the occurrence of sensitization to various classes of allergens depending on patients’ age [55]. Profiles of the levels of allergen-specific sIgE and sIgG4 in each specimen were obtained. Among inhaled allergens, sensitization was most frequently caused by birch pollen and cat dander, while the sIgE response was typically related to egg and milk allergens, among food allergens (*Fig. 12*). The percentage of patients with elevated levels of sIgE specific to inhaled allergens increases with age, while the percentage of patients sensitized to most of the food allergens (except for carrot, apple, and peach allergens) decreases.

## CONCLUSIONS

It has been 30 years since the first study focused on sequencing by hybridization with immobilized octanucleotide probes was published [56]. During this period, researchers at the EIMB Laboratory of Biological Microarrays have developed universal methods to be used for multi-parameter analysis of protein and DNA markers in statistical and clinical studies involving large series of biological specimens of differing nature. A production line to manufacture hydrogel-based microarrays, with an annual capacity of up to 1 million microarrays, has been established and certified as in compliance with the international standard ISO 13485. The Russian Federal Service for Surveillance in Healthcare and Social Development has granted 12 registration certificates to the EIMB for the developed medical devices (reagent kits and equipment for in vitro diagnostics using hydrogel-based microarrays).

With PCR technologies and next-generation sequencing platforms rapidly evolving, DNA microarrays have recently faced serious competition. Today, DNA microarrays occupy an intermediate niche between various nucleic acid amplification tests attempting to outcompete high-throughput sequencing technologies and exerting growing pressure. In our case, immobilization of any types of biomolecules in hydrogel and the feasibility of conducting enzymatic reactions in it [57], including isothermal ones [58], makes it possible to design portable next-generation biosensors. Hence, 3D hydrogel elements can be used as a platform for simultaneous immobilization of genome-editing agents (the nucleases Cas13 and Cas12a), together with guiding and detecting RNA/DNA molecules [59]. The “programmable” performance of nucleases (if needed, supplemented with isothermal amplification), in combination with the elaborated microfluidic systems for the isolation of nucleic acids [60], will allow the manufacture of highly sensitive CRISPR-biosensors. These biosensors could potentially be used under field conditions. These integrative autonomous systems containing the hydrogel-based microarray platform and the coordinated supporting modules will make it possible to obtain more informative and accurate results in a shorter time than is now the case. They will play a crucial role in the personalized medicine of the future.

Thus far, more than 2,000 patients of the Filatov Moscow Pediatric Clinical Hospital have been examined using the Allergo-biochip kit. In addition to the apparent economic benefit due to the “one specimen– one analysis” format, the proposed approach allows one to identify the allergen causing a severe allergic reaction in a child using only 100 µL of serum. Such a small specimen volume is a substantial advantage when examining children of a young age (several months old). It is rather promising to design protein microarrays for the differential diagnosis of rheumatologic diseases and other immune disorders. Extending the scope of microarray applications to the analysis of predictive markers for cancer also holds great promise. It is our hope that developing a new approach based on comprehensive signature analysis will allow clinicians to solve this challenging problem.

Hence, the hydrogel microarray technology has already proved an efficient tool in personalized medicine. It allows one to perform molecular profiling of a plethora of clinically significant markers of causative agents and reasons for socially important diseases, save the lives of hundreds of patients, and optimize the public funds allocated for healthcare.

## Abbreviations

NAs: nucleic acids
NGS: next-generation sequencing
FDA: Food and Drug Administration (USA)
MTB: Mycobacterium tuberculosis, causative agent of tuberculosis
NTM: non-tuberculous mycobacteria, causative agents of mycobacteriosis
HCV: hepatitis C virus
RMP: rifampin
INH: isoniazid
EMB: ethambutol
MDR: multidrug resistance
XDR: extensive drug resistance
RTIs: reproductive tract infections
AMD: antimicrobial drugs
CRC: colorectal carcinoma
CEA: carcinoembryonic antigen
CA: carbohydrate antigen
